# Irinotecan toxicity: genes or intestinal microflora?

**DOI:** 10.1038/sj.bjc.6604957

**Published:** 2009-03-17

**Authors:** G Brandi, F de Rosa, G Biasco

**Affiliations:** 1 Department of Haematology and Medical Oncology ‘L. e A. Seràgnoli’, University of Bologna, Bologna, Italy

**Sir,**

As research advances, a growing body of evidence shows that the individual response to any drug, i.e. effectiveness and toxicity, may be influenced by genetic polymorphisms. In the case of anticancer chemotherapeutic agents, characterised by a low therapeutic index, pharmacogenomic studies could stratify patients for expected response and adverse effects, potentially allowing more rational prescriptions.

Among various drugs, irinotecan has been extensively studied. Actually it is a prodrug affected by two main dose-limiting toxicities: neutropoenia and delayed diarrhoea. The recent work by Rouits *et al* evaluates the role of the polymorphisms of two enzymes involved in irinotecan metabolism: UGT1A1, responsible for glucuronisation and disposal of its active metabolite SN-38; carboxylesterase-2 (CES2), responsible for SN-38 formation. CYP3A4 activity, responsible for irinotecan transformation into the inactive compounds APC and NPC, has been indirectly estimated by urinary 6*β*-hydroxycortisol/cortisol ratio, and can be influenced by many ambiental factors (Rouits *et al*, 2008).

As authors say, UGT1A1 variants affect only haematological toxicity. An earlier, larger trial had similar results, but was statistically significant only for the first cycle and did not correlate with necessity of dose reduction (Toffoli *et al*, 2006). Carboxylesterase-2 variants are not significantly correlated with any parameter, whereas urinary 6*β*-hydroxycortisol/cortisol ratio seemed as a global predictor of toxicity including diarrhoea, but it could simply reflect hepatic dysfunction and therefore needs further evaluation.

These results are somewhat disappointing. Chemotherapy-induced neutropoenia can be easily managed with recombinant myeloid growth factors if necessary, whereas diarrhoea cannot be reliably predicted and is sometimes severe. The complex pharmacology of irinotecan can actually be influenced by many factors, both genetic and nongenetic. In particular its action on the intestinal mucosa is strongly influenced by local environmental factors, especially intestinal microflora.

Actually SN-38 glucuronide, excreted in faeces, can be processed by bacterial *β*-glucuronidase and converted back to SN-38, which can damage intestinal epithelial cells and thus induce delayed diarrhoea. This hypothesis have been suggested by Takasuna *et al* who showed that intestinal damage due to irinotecan administration is more pronounced in gut segments with high *β*-glucuronidase activity (Takasuna *et al*, 1996).

A subsequent study specifically aimed at investigating the role of intestinal microflora in irinotecan-induced diarrhoea compared holoxenic and germ-free mice. Irinotecan lethal dose is more than twice higher in the second group, which also exhibited significantly less diarrhoea. Intestinal damage as shown by histological examination reflected bacterial distribution along the gut, in particular it was negligible in germ-free animals treated at 60 mg kg^−1^ d^−1^ for 4 days, the lethal dose for holoxenic controls (Brandi *et al*, 2006).

The poor predictive value for diarrhoea of the genetic loci studied by Rouits *et al* is therefore not surprising. In humans, intestinal microflora composition is quite stable along life and host-specific, representing a sort of adjunctive fingerprint. Consequently, *β*-glucuronidase activity characteristic of some bacterial strains only, can be quite changeable among various subjects. Both factors (genetic polymorphisms and intestinal microflora) can influence irinotecan intestinal toxicity: this constitutes the rationale for the necessity of evaluating also intestinal microflora and *β*-glucuronidase role in future studies investigating its interindividual variability.

## References

[bib1] Brandi G, Dabard J, Raibaud P, Di Battista M, Bridonneau C, Pisi AM, Morselli Labate AM, Pantaleo MA, De Vivo A, Biasco G (2006) Intestinal microflora and digestive toxicity of irinotecan in mice. Clin Cancer Res 12: 1299–1307 16489087 10.1158/1078-0432.CCR-05-0750

[bib2] Rouits E, Charasson V, Pétain A, Boisdron-Celle M, Delord JP, Fonck M, Laurand A, Poirier AL, Morel A, Chatelut E, Robert J, Gamelin E (2008) Pharmacokinetic and pharmacogenetic determinants of the activity and toxicity of irinotecan in metastatic colorectal cancer patients. Br J Cancer 99: 1239–1245 18797458 10.1038/sj.bjc.6604673 PMC2570505

[bib3] Takasuna K, Hagiwara T, Hirohashi M, Kato M, Nomura M, Nagai E, Yokoi T, Kamataki T (1996) Involvement of beta-glucuronidase in intestinal microflora in the intestinal toxicity of the antitumor camptothecin derivative irinotecan hydrochloride (CPT-11) in rats. Cancer Res 56: 3752–3757 8706020

[bib4] Toffoli G, Cecchin E, Corona G, Russo A, Buonadonna A, D'Andrea M, Pasetto LM, Pessa S, Errante D, De Pangher V, Giusto M, Medici M, Gaion F, Sandri P, Galligioni E, Bonura S, Boccalon M, Biason P, Frustaci S (2006) The role of UGT1A1^*^28 polymorphism in the pharmacodynamics and pharmacokinetics of irinotecan in patients with metastatic colorectal cancer. J Clin Oncol 24: 3061–3068 16809730 10.1200/JCO.2005.05.5400

